# A Retrospective Analysis of Real-Life Management of Colorectal Cancer Lung-Limited Metastases Treated with Surgery: Outcomes and Prognostic Factors

**DOI:** 10.3390/jcm13226651

**Published:** 2024-11-06

**Authors:** Ina Valeria Zurlo, Maria Alessandra Calegari, Maria Teresa Congedo, Michele Basso, Maria Letizia Vita, Leonardo Petracca Ciavarella, Raffaella Vivolo, Annunziato Anghelone, Carmelo Pozzo, Lisa Salvatore, Elisa Meacci, Stefano Margaritora, Giampaolo Tortora

**Affiliations:** 1 Oncologia Medica, Fondazione Policlinico Universitario “A Gemelli”—IRCCS, Largo Francesco Vito n 1, 00168 Rome, Italy; mariaalessandra.calegari@policlinicogemelli.it (M.A.C.); michele.basso@policlinicogemelli.it (M.B.); raffaella.vivolo@unicatt.it (R.V.); annunziato.anghelone@ospedalerc.it (A.A.); carmelo.pozzo@policlinicogemelli.it (C.P.); lisa.salvatore@policlinicogemelli.it (L.S.); giampaolo.tortora@policlinicogemelli.it (G.T.); 2 Oncologia Medica, Università Cattolica del Sacro Cuore, 00168 Rome, Italy; 3 Unità di Chirurgia Toracica, Fondazione Policlinico Universitario “A Gemelli”—IRCCS, 00168 Rome, Italy; mariateresa.congedo@policlinicogemelli.it (M.T.C.); marialetizia.vita@policlinicogemelli.it (M.L.V.); leonardo.petraccaciavarella@policlinicogemelli.it (L.P.C.); elisa.meacci@policlinicogemelli.it (E.M.); stefano.margaritora@policlinicogemelli.it (S.M.); 4 Unità di Chirurgia Toracica, Università Cattolica del Sacro Cuore—IRCCS, 00168 Rome, Italy

**Keywords:** colorectal cancer, lung surgery, multidisciplinary management

## Abstract

**Background**: Unlike liver metastases, the role of surgery in colorectal cancer lung-limited metastases (CCLLM) is not yet established, and data are still poor. We performed a retrospective analysis to evaluate the impact of surgery on the management of CCLLM. **Material and Method**: We retrospectively analyzed patients who received surgery for CCLLM at our Institution from January 2010 to June 2019. The aim of the study was to evaluate the impact of clinical and pathological features on the survival (OS and DFS) of patients treated with surgery for CCLLM. **Results**: One hundred and fifty patients were included in the analysis. Seventy-six patients received preoperative chemotherapy (pCT) and 56 an adjuvant treatment (aCT), while 18 underwent up-front surgery without CT. In the whole population, median OS (mOS) and median DFS (mDFS) were 54.1 months (95%CI 44.0–82.1) and 24.0 months (95%CI 20.0–31.2), respectively. In multivariate analysis, number of metastases was the only factor correlated to DFS (*p* = 0.0006) and OS (*p* = 0.0018). **Conclusion**: Our study, although retrospective and of small size, shows that tumor burden (number of metastases) is the main prognostic factor in patients undergoing lung surgery for CCLLM. Moreover, our results suggest that surgery for lung metastases might prolong survival. These data strengthen the role of multidisciplinary management to allow patients with CCLLM to pursue local treatment whenever possible, even regardless of previous liver surgery or *RAS* mutated status.

## 1. Introduction

Colorectal cancer (CRC) is the third most common cancer worldwide [1]. About 50% of patients with CRC are diagnosed at late stages, which significantly reduces the possibility of being cured and the availability of treatment options other than chemotherapy (CT). Moreover, even localized disease will develop metastases in approximately 20–30% of cases. The liver and lungs are the organs most commonly affected as metastatic sites [2], respectively. About 10–15% of patients show lung metastases at diagnosis or will develop them later [3]. Moreover, an average of 2.3% of patients receive pulmonary metastasectomy (PM) for CRC lung-limited metastasis (CCLLM) [3].

Although the role of surgery for colorectal liver metastases is well-established, with promising 5-year and 10-year survival rates (58% and 25–28%, respectively), evidence supporting surgery for lung metastases is weak. Indeed, although surgery experiences of colorectal cancer lung-limited metastases (CCLLM) have increased in the last years, data are retrieved mainly from monocentric [4,5,6] and multicentric [7] retrospective studies or from systematic reviews [8,9]. Reported 5-year overall survival (OS) rate ranges from 27–68% [4,5,6,7,8,9]. Moreover, several retrospective analyses showed improved survival rates after both liver and lung resections, with 5-year survival rates of 30–70% [10,11,12]. More recently, a Pulmonary Metastasectomy in Colorectal Cancer (PulMiCC) study investigated the impact of this practice in a prospective trial [13] and in a randomized controlled non-inferiority trial [14]. This study confirmed the survival rates previously observed in single-arm studies. Interestingly, patients enrolled in the control groups displayed greater survivals than expected (ranging between 22–29%).

Despite the lack of evidence of randomized studies showing the benefit of PM over CT alone, international guidelines recognize lung surgery as a potentially curative treatment and recommend resections as a standard strategy to improve prognosis whenever feasible [15]. Indeed, for oligometastatic disease (OMD) confined to a single organ or a few organs, surgery is the only potentially curative option.

Nevertheless, since mainly retrospective studies and surgical reports are available and prospective data are still poor, the management of CCLLM still remains an open debate [12,16].

In the last decade, many improvements have been made in the management of metastatic CRC (mCRC) as a result of novel surgical techniques, advances in systemic treatments, and the establishment of multidisciplinary assessment, allowing room for surgery even in advanced scenarios where, up to a few years ago, palliative CT was the only option [17,18].

Concerning systemic treatment, the introduction of biological agents and patients’ selection based on molecular features, leading to tailored therapy, played a huge role in improving patients’ outcomes, allowing more aggressive treatment strategies and sequential liver and lung resections in OMD. Nevertheless, relapses are still a challenge and range from 20 to 68% [19,20,21,22].

Many retrospective series attempted to identify prognostic factors to guide clinical decisions, with particular regard to investigating the role of mediastinal lymph node involvement, large metastases, high preoperative serum CEA levels, and timing of metastases onset. These reports obtained conflicting results [7,20,23,24,25]. Some evidence showed that *KRAS* mutations are strong prognostic predictors for poor survival in mCRC patients undergoing PM [26]. Other reports seemed to exclude a poor prognostic role for *RAS* mutations, instead confirming a worse prognosis only for *BRAF* mutations [27,28,29]. Lastly, the role of systemic CT for resectable CCLLM, either perioperative or adjuvant, is still controversial with few data concerning its impact on survival [7,30,31,32,33,34,35].

We performed this retrospective analysis to evaluate outcomes of CCLLM treated with surgery at our center, exploring prognostic factors affecting survival.

## 2. Materials and Methods

This is an observational, retrospective, mono-institutional study. The study was approved by the local Ethics Committee of Fondazione Policlinico Universitario Agostino Gemelli, IRCCS, Rome, Italy (protocol number 0054049/2019 18 December 2019) and was conducted in accordance with the Declaration of Helsinki. The clinical records of patients diagnosed with only CCLLM who were treated at our institution between January 2010 and June 2019 were reviewed. Our analysis also includes a subgroup of patients who had lung metastases along with resectable liver metastases at baseline, as well as those who later developed resectable or potentially resectable liver metastases. All patients’ data were collected anonymously. Informed consent was obtained from all subjects and/or their legal guardian(s).

The objective of the study was to analyze the impact of clinical and pathological features on survival outcomes of patients with CCLLM treated with pulmonary metastasectomy (PM). The primary end-point was overall survival (OS). The secondary end-point was disease-free survival (DFS). OS was defined as the time from PM to the date of death due to any cause or was censored at the date of the last follow-up for patients still alive. DFS was defined as the time from PM to the date of first documented recurrence (either local, regional, or distant) or death due to any cause, whichever occurred first. The Kaplan–Meier method was used to estimate OS and DFS. A Cox regression model was employed to estimate hazard ratios (HRs). The statistical significance level was set at *p* < 0.05. Univariate analysis was performed to establish the relationship between survival endpoints and clinicopathological features: Age (<65 vs. >65 years), gender (male vs. female), timing of lung metastases onset (metachronous vs. synchronous), disease-free interval (DFI) between primary colorectal surgery and lung recurrence (>18 vs. <18 months), primary tumor location (right vs. left), mucinous histology (present vs. absent), grading (G1–G2 vs. G3), RAS mutational status (mutated vs. wild-type), number of metastases (1 vs. ≥2), metastases location (one-sided vs. bilateral), exposure to perioperative chemotherapy (pCT) or adjuvant CT (aCT) (yes vs. no), and liver resection performed for resectable liver metastases. Synchronous pulmonary metastasis was defined as the development of a lung lesion within 6 months from primary tumor resection.

Differences were compared with the use of a log-rank test and statistically significant (*p* value < 0.5) parameters at univariate analysis were included in the multivariate analysis.

Data were analyzed using MedCal Statistical software version number 23.0.6.

## 3. Results

Between January 2010 and June 2019, 150 patients underwent PM for CCLLM with curative intent at our institution and were included in the analysis. Clinicopathological characteristics are shown in Table 1. The median age was 61 years (range 38–80 years). Eighty-three patients (55.3%) were male and 67 (44.7%) were female. All patients had a performance status (PS) of 0–1. The primary tumor was mainly located on the left side (122 patients; 81.4%) rather than on the right side (28 patients; 18.6%). Twenty-two patients (14.6%) had a mucinous histology. Twenty-nine patients (19.3%) had a poorly differentiated tumor, and 121 patients (80.7%) had a moderately differentiated tumor. One hundred twenty-two patients (81.3%) had metachronous metastases, while 28 (18.7%) had a synchronous disease. Seventy-two patients (48%) also received liver surgery, 56 before and 16 after PM. Of those, the disease was metachronous in 45 patients and synchronous in 27 patients. Patients with metachronous liver metastases underwent liver surgery before PM, with lung metastases detected during follow-up, at which point they had only thoracic disease. In contrast, patients with synchronous disease who underwent PM before liver surgery developed liver metastases afterward. All these patients were included in the analysis, as they had no other disease sites at the time of thoracic surgery. OS and DFS were measured from the time of PM to death and relapse in both cases, respectively. Median number of lung metastases was 1 (range 1–8). Ninety patients (60%) had a single metastasis, whereas sixty patients (40%) showed multiple lung metastases (>1). One hundred sixteen patients (77.3%) had disease on only one side, while 34 patients (22.7%) had disease on both sides.

*RAS* mutational status, including both *KRAS* and *NRAS* exons 2, 3, and 4 analysis, and *BRAF* mutational status, including codon 600, have been evaluated in 116 patients (77.3%). Of these, 57 were wild-type and 59 were *KRAS* or *NRAS* mutated. No *BRAF* mutation was found. No information concerning microsatellite status or mismatch repair protein expression was available.

Overall, 100 wedge resections (67.3%), 27 segmentectomies (18%), and 22 lobectomies (14.7%) were performed. All patients received R0 resections. Seventy-six patients (50.7%) received pCT and 56 (37.3%) received aCT, while 18 (12%) underwent up-front surgery without any CT. Perioperative regimens administered included: FOLFOX (36 patients; 24%), FOLFIRI plus bevacizumab (20 patients; 13.3%), FOLFOX plus bevacizumab (5 patients; 3.3%), FOLFIRI plus aflibercept (1 patient; 0.7%), and FOLFIRI plus cetuximab (14 patients; 9.3%). Of note, four patients received two prior lines before PM. FOLFOX and capecitabine have been administered as adjuvant treatments in 46 (30.7%) and 10 (6.7%) patients, respectively. Ten patients underwent further PM for later recurrence.

**Figure 1:**
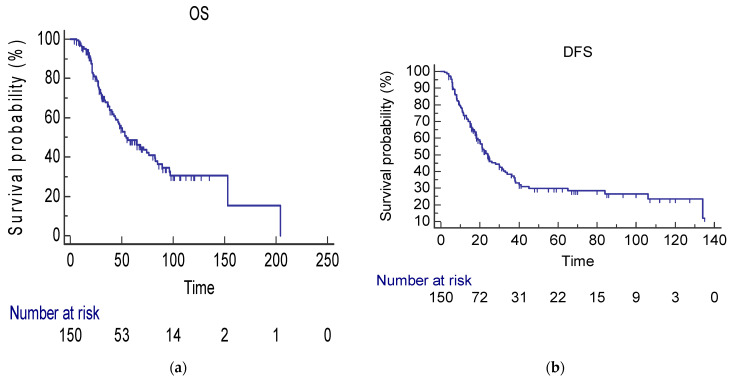
(**a**) mOS in the whole population (54.1 months; 95%CI 44–82.11 months). CI confidence interval, mOS median overall survival. (**b**) mDFS in the whole population (24 months; 95%CI 20–31.2 months). CI confidence interval, mDFS median disease-free survival.

**Figure 2:**
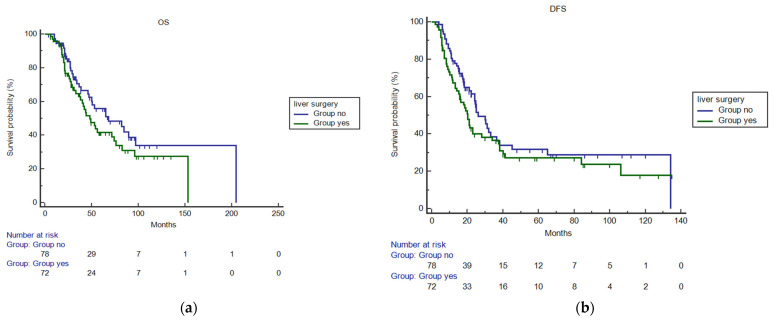
(**a**) mOS in patients who received lung surgery only compared to those treated with liver and lung surgery (HR 1.37, 95%CI 0.86–2.1; *p* = 0.17). (**b**) mDFS in patients who received lung surgery only compared to those treated with liver and lung surgery (HR 1.32, 95%CI 0.88–1.98; *p* = 0.16).

At a median follow-up of 33 months (range 6–153 months), 73 events for OS and 95 events for DFS were observed. In the whole population (150 patients), the median OS (mOS) was 54,1 months (95%CI 44.0–82.1 months) (Figure 1a). Median DFS (mDFS) was 24.0 months (95%CI 20.0–31.2 months) (Figure 1b). Among patients who received both liver and lung surgery (72 patients), according to the characteristics mentioned above, mOS and mDFS were 53.0 months (95%CI 40.09–82.11 months) and 21 months (95%CI 16–34.0 months), respectively. Among patients who underwent only lung surgery mOS was 65 (95%CI 46–97,009) and mDFS 26 months (95%CI 21.0–36, months). When comparing patients who received lung surgery only (78 patients) to those who received both liver and lung surgery (72 patients), no statistical significant difference was observed for both mOS (HR 1.37, 95%CI 0.86–2.1; *p* = 0.17) (Figure 2a) and mDFS (HR 1.32, 95%CI 0.88–1.98; *p* = 0.16) (Figure 2b).

Age, gender, timing of lung metastases onset, DFI, primary tumor location, mucinous histology, grading, *RAS* mutational status, number of metastases, metastases location, pCT or aCT, and liver resection were evaluated at univariate and multivariate analysis. At univariate analysis, DFI > 18 m (*p* = 0.042), bilateral metastases location (*p* = 0.0061), a higher number of metastases (*p* < 0.0001), and pCT (*p* = 0.016) correlated with DFS (Table 2). Whereas OS was associated only with the number of metastases (*p* = 0.0001) (Table 2). At multivariate analysis, number of metastases was the only factor correlated to DFS (*p* = 0.0006) and OS (*p* = 0.0018) (Table 3). In the subgroup of patients who received both lung and liver surgery, timing of lung metastases onset, number of lesions, and *RAS* mutational status correlated with OS at univariate analysis (*p* = 0.044, *p* = 0.0077 and *p* = 0.02, respectively). No clinicopathological characteristic retained its prognostic impact on OS at multivariate analysis (Table 4).

## 4. Discussion

Although strong evidence for surgery in oligometastatic lung disease is still limited, this approach is widely recognized and recommended in international guidelines, partly because pulmonary metastasectomy has low mortality and complication rates [2,7]. Since isolated lung metastases are uncommon, the decision to proceed with lung surgery often depends on CT scan results. Moreover, studies indicate a survival benefit for patients who undergo lung resection after liver resection—approximately 29% of patients receiving lung surgery fall into this category [36,37]. Given these considerations, multidisciplinary assessment is essential, as a team approach is more likely to lead to curative treatment strategies and improve survival outcomes.

Maybe the main finding of our study is that tumor burden (assessed as a number of lesions) represents the main prognostic factor for patients undergoing lung surgery for CCLLM, impacting both DFS and OS. Whereas no correlation was observed between metastases location (monolateral or bilateral) or *RAS* mutational and survivals at multivariate analysis. These results are consistent with other retrospective studies, although it remains challenging to determine a specific number of lung metastases that could impact outcomes [38].

Our cohort showed an mOS longer than 50 months, which is largely superior to the survival of patients treated in a metastatic setting within large randomized phase III studies. Moreover, the mOS of patients receiving both liver and lung surgery are quite similar, with no feature affecting outcomes in this subgroup at multivariate analysis. This evidence reinforces the need for detailed assessment and discussion within a multidisciplinary team in order to ensure that a surgical strategy is taken into account, following careful patient selection whenever an R0 resection is possible. Indeed, surgery is the standard treatment for OMD, being the only potentially curative option and several reports confirm the benefit of surgery for both lung and liver metastasis [15,39,40]. When resection is limited by comorbidity, extent of lung parenchyma resection, or other factors, other local ablative treatments (LATs; such as stereotactic radiotherapy [41] or local thermal ablation) should be considered. LATs could be combined with systemic CT, as part of a multimodal therapy approach, in order to achieve long-term disease control, potentially contributing to survival.

Similar to Fong’s criteria for liver metastasis, some studies aim to establish a clinical prognostic score to improve patient selection for surgery. The five prognostic variables of the Meta-Lung score, identified by Ziranu et al. [42], may assist clinicians in selecting patients with a better prognosis who are more likely to benefit from thoracic surgery for PM.

Currently, the PUCC trial is investigating the role of surgery versus standard chemotherapy in CRC patients with poorer prognoses and ≥ 3 CCLLM [43].

Finally, new biomarkers—such as radiomics, immunoscore, and ct-DNA—are essential for better risk stratification and identifying patients at high risk for CRC recurrence after surgical metastasectomy [44,45].

Our study has some limitations. Firstly, given the retrospective nature of the analysis, a selection bias is possible. In fact, the prevalence of left-sided tumors (81%), the low rate of mucinous histology and poorly differentiated cancers (about 20%), and the high prevalence of metachronous disease and monolateral lung involvement suggest a good prognosis population. Interestingly, survivals observed in the control arms without metastasectomy of the PulMiCC study [13,14] displayed greater survivals than expected (ranging between 22–29%), confirming that this population is enriched with positive prognostic features. Indeed, OMD could be considered a unique biological entity with a favorable prognosis [46].

Nevertheless, other factors could have contributed to the positive findings observed in terms of both DFS and OS. Systemic CT, administered in 88% of patients either in adjuvant or peri-operative settings, could have reduced the risk of relapse, prolonging the time of recurrence (as confirmed by the impact of pCT on DFS at univariate analysis). However, no conclusive inference can be drawn on this topic. Moreover, since about 50% of patients received liver surgery in addition to lung surgery, also the role of the multidisciplinary assessment seems to be crucial for the positive outcomes of our patients.

Other limitations of the study rely on its single-institution nature, the small sample size, and the long period of analysis (2010–2019), during which therapeutic strategies, molecular assessment, and drugs have changed. Moreover, the analysis lacks a control arm.

Despite those limitations, our results are consistent with previous evidence and clearly suggest that surgery with radical intent, whenever possible, might prolong survival. Finally, these data strengthen the role of multidisciplinary management to allow patients with CCLLM to pursue local treatment whenever possible. These data need to be confirmed in larger and prospective series.

## Figures and Tables

**Table 1:** Patients’ characteristics.

	*n*	%
**Gender**		
Male	83	55.3
Female	67	44.7
**Primary tumor location**		
Right	28	18.6
Left/rectum	122	81.4
**Mucinous histology**	22	14.6
**Grading**		
G3	29	19.3
G2	121	80.7
**Liver surgery**	72	48
**Metastases location**		
One-sided	116	77.3
Bilateral	34	22.7
**Type of surgery**		
Wedge resection	100	67.3
Segmentectomies	27	18
Lobectomies	22	14.7
***RAS* and *BRAF* mutational status**	116	
*KRAS*/*NRAS* mt	59	39.3
*RAS*/*BRAF* wt	57	38
Unknown	34	22.7
**Perioperative CT**	76	50.7
Folfox	36	24
Folfiri + bevacizumab	20	13.3
Folfox + bevacizumab	5	3.3
Folfiri + aflibercept	1	0.7
Folfiri + cetuximab	14	9.3
**Adjuvant CT**	56	37.3
Folfox	46	30.7
Capecitabine	10	6.7
**Surgery alone**	18	12

CT, chemotherapy; *n*, number; mt, mutated; wt, wild type.

**Table 2:** Univariate analysis for OS and DFS on the whole population.

Age		
<65 vs. >65	1.2 (0.66–1.57) 0.91	1.2 (0.66–1.57) 0.91
Gender		
male vs. female	1.09 (0.73–1.64) 0.65	1.09 (0.73–1.64) 0.65
Grading		
G1–2 vs. G3	1.4 (0.79–2.542) 0.19	1.4 (0.79–2.542) 0.19
Mucinous histology		
present vs. absent	1.26 (0.69–2.29) 0.40	1.26 (0.69–2.29) 0.40
Number of metastases		
(1 vs. ≥2)	2.7 (1.44–5.06) <0.0001	2.7 (1.44–5.06) <0.0001
Metastases location		
one-sided vs. bilateral	0.53 (0.30–0.93) 0.0061	0.53 (0.30–0.93) 0.0061
Primary tumor location		
right vs. left	1.02 (0.60–1.73) 0.92	1.02 (0.60–1.73) 0.92
*RAS* mutational status		
mutated vs. wild-type	1.17 (0.74–1.84) 0.47	1.17 (0.74–1.84) 0.47
DFI		
>18 vs. <18 months	1.51 (1.006–2.26) 0.04	1.51 (1.006–2.26) 0.04
Liver resection		
yes vs. no	1.32 (0.88–1.98) 0.167	1.32 (0.88–1.98) 0.167
Timing of lung metastases onset		
metachronous vs. synchronous	1.51 (0.929–2.47) 0.06	1.51 (0.929–2.47) 0.06
pCT		
yes vs. no	1.67 (1.11–2.50) 0.016	1.67 (1.11–2.50) 0.016
aCT		
yes vs. no	1.27 (0.84–1.92) 0.23	1.27 (0.84–1.92) 0.23

aCT, adjuvant chemotherapy; CI, confidence interval; DFI, disease-free interval; DFS, disease-free survival; HR, hazard ratio; OS, overall survival; pCT, perioperative chemotherapy.

**Table 3:** Multivariate Cox regression analysis for OS and DFS for clinicopathological variables resulted in significant univariate analysis in the whole population.

	DFS		OS	
	HR (95%CI) for Progression	*p* Value	HR (95% CI) for Mortality	*p* Value
DFI				
>18 vs. <18 months	1.49 (0.97–2.27)	0.0663	1.41 (0.74–2.70)	0.29
Metastases location				
one-sided vs. bilateral	0.94 (0.52–1.70)	0.8586	2.39 (0.91–6.25)	0.07
Number of metastases				
(1 vs. ≥2)	2.74 (1.55–4.8)	0.0006	4.59 (1.77–11.9)	0.0018
pCT				
yes vs. no	1.45 (0.93–2.26)	0.1002	1.09 (0.55–2.14)	0.79

CI, confidence interval; DFI, disease-free interval; DFS, disease-free survival; HR, hazard ratio; OS, overall survival; pCT, perioperative chemotherapy.

**Table 4:** Univariate and multivariate analysis for OS in the subgroup of patients who received both lung and liver metastases resection.

	Univariate OS		Multivariate OS	
	HR (95%CI) for Mortality	*p* Value	HR (95% CI) for Mortality	*p* Value
Age	1.05 (0.52–2.11)	0.88	0.86 (0.17–4.19)	0.85
Gender	0.79 (0.42–1.47)	0.45	0.81 (0.08–4.49)	0.65
Grading				
G1-2 vs. G3	1.21 (0.50–2.89)	0.65	1.13 (0.20–6.21)	0.176
Mucinous histology present vs. absent	1.89 (0.79–3.24)	0.14	3.8 (0.63–3.97)	0.77
Number of metastases	3.21 (1.11–9.3)	0.0007	2.4 (1.4–8.3)	0.32
Metastases location	0.78 (0.35–1.74)	0.51	2.23 (0.46–10.6)	0.31
Primary tumor location				
right vs. left	1.06 (0.51–2.40)	0.86	1.19 (0.19–7.36)	0.84
DFI >18 vs. <18 months	0.79 (0.42–1.47)	0.45	4.99 (0.75–3.08)	0.097
Timing of lung metastases onset				
metachronous vs. synchronous	1.98 (0.98–4.00)	0.044	2.36 (0.23–4.08)	0.46
pCT				
yes vs. no	1.06 (0.47–2.36)	0.88	3.41 (0.29–4.91)	0.32
aCT	0.99 (0.52–1.89)	0.99	0.17 (0.3–1.75)	0.20
*RAS* mutational status				
mutated vs. wild-type	2.18 (1.04–4.57)	0.02	4.09 (0.53–3.37)	0.17

CI, confidence interval; DFI, disease-free interval; HR, hazard ratio; OS, overall survival; pCT, perioperative chemotherapy.

## Data Availability

The datasets used and/or analyzed during the current study are available from the corresponding author upon reasonable request.
